# Development of B Cells and Erythrocytes Is Specifically Impaired by the Drug Celastrol in Mice

**DOI:** 10.1371/journal.pone.0035733

**Published:** 2012-04-24

**Authors:** Sophie Kusy, Eliver E. B. Ghosn, Leonore A. Herzenberg, Christopher H. Contag

**Affiliations:** 1 Department of Pediatrics, Stanford University School of Medicine, Stanford, California, United States of America; 2 Department of Genetics, Stanford University School of Medicine, Stanford, California, United States of America; 3 Department of Radiology, Stanford University School of Medicine, Stanford, California, United States of America; 4 Department of Microbiology and Immunology, Stanford University School of Medicine, Stanford, California, United States of America; 5 Molecular Imaging Program at Stanford (MIPS), Stanford University School of Medicine, Stanford, California, United States of America; Heart Center Munich, Germany

## Abstract

**Background:**

Celastrol, an active compound extracted from the root of the Chinese medicine “Thunder of God Vine” (*Tripterygium wilfordii*), exhibits anticancer, antioxidant and anti-inflammatory activities, and interest in the therapeutic potential of celastrol is increasing. However, described side effects following treatment are significant and require investigation prior to initiating clinical trials. Here, we investigated the effects of celastrol on the adult murine hematopoietic system.

**Methodology/Principal Findings:**

Animals were treated daily with celastrol over a four-day period and peripheral blood, bone marrow, spleen, and peritoneal cavity were harvested for cell phenotyping. Treated mice showed specific impairment of the development of B cells and erythrocytes in all tested organs. In bone marrow, these alterations were accompanied by decreases in populations of common lymphoid progenitors (CLP), common myeloid progenitors (CMP) and megakaryocyte-erythrocyte progenitors (MEP).

**Conclusions/Significance:**

These results indicate that celastrol acts through regulators of adult hematopoiesis and could be used as a modulator of the hematopoietic system. These observations provide valuable information for further assessment prior to clinical trials.

## Introduction

*Tripterygium wilfordii*, an ivy-like vine also known as the “Thunder God Vine”, has been used as natural medicine in China for hundreds of years [1]. Celastrol, a quinone methide triterpenoid, was identified to be one of its active components. As root extract or purified compound, its remarkable anti-inflammatory ability has been demonstrated in animal models of different inflammatory diseases including asthma [2], Crohn's disease [3], and neurodegenerative disorders [4], [5]. Purified celastrol showed anticancer activity, in vivo in various tumor models of melanoma [6], prostate [7] and breast [8] cancer, as well as in vitro on leukemic cell lines [9], [10], [11], suggesting its use as a cancer therapeutic.

However, multiple side effects have been reported, including leukopenia, thrombocytopenia and anemia [12]. These adverse reactions are transient and recovery is usually complete upon removal of the drug. The molecular bases of the therapeutic and side effects are not well understood. Therefore, to advance celastrol as a therapeutic and prevent side effects, its toxicity and mechanism of action need to be revealed.

In the present study, we investigated the effects of celastrol on the hematopoietic system of adult mice with the aim of describing the immediate effects of celastrol on different mature and progenitor hematopoietic cell populations. We observed significant alterations of stem cells, progenitors and fully differentiated cell populations in peripheral blood (PB), bone marrow (BM), spleen and peritoneal cavity (PerC). These data indicate significant hematotoxicity, and suggest differential effects of celastrol on specific hematopoietic subsets. Understanding these effects will better enable the use of this potential therapeutic agent and will identify new clinical applications.

## Materials and Methods

### 1- Mice and chemical treatment

BALB/c mice, 8- to 10-weeks-old, were purchased from Jackson Labs. All mice were maintained in the Animal Facility at Stanford University School of Medicine. All experiments were conducted under strict adherence to institutional guidelines, as approved by the Animal Care and Use Committee at Stanford University (aplac #12323). Mice received daily intraperitoneal (IP) injections (200 µL) of either celastrol (Cayman Chemical, purity ≥ 98%) diluted solutions (0.01, 0.1, 1 or 5 mg/kg/day), or carrier only (PBS with 5% DMSO) as a control, over the course of four days.

### 2- Tissue preparation and flow cytometry

Tissues were harvested the day following the last injection as previously described [13]. Cells were counted and stained with antibodies for phenotyping. For mature population analyses, cell suspensions were preincubated with anti–CD16/CD32 mAb to block FcγRII/III receptors and stained with the following fluorochrome-conjugated mAb: FITC-labeled anti-CD71; PE-labeled anti–Ter119, PECy5-labeled anti-CD5; PECy5.5-labeled anti-CD19; PECy7-labeled anti–IgM; APCCy5.5-labeled anti-IgD; APCCy7-labeled anti-CD11b and Pacific Blue–labeled anti–Gr-1, and Violet Green-labeled LIVE/DEAD®. For progenitors analyses, cells were stained with FITC-labeled anti-CD34, PE-labeled anti–Sca1, APC-labeled anti-c-Kit, PECy7-labeled anti-Il7Ra, APCCy7-labeled anti-CD16/32 and PerCPCy5.5-labeled lineage antibody cocktail. Antibodies were either purchased (Invitrogen and BD) or conjugated in the Herzenberg laboratory. Cells were analyzed on LSRII (BD). Data were analyzed with FlowJo software (TreeStar). In the carrier only treated-animals (PBS with 5% DMSO) there were no detectable changes in the levels of CD expression.

### 3- Statistical analyses

Quantitative data are expressed as the mean ± SEM. Statistical significance was assayed using a non-parametric Mann Whitney test (n = 10 mice. *p<0.05; **p<0.01; ***p<0.001).

## Results and Discussion

### 1- Changes in peripheral blood parameters in celastrol treated-mice

**Figure 1:**
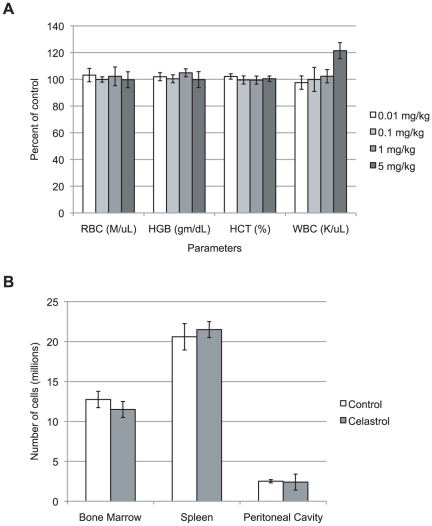
Changes in peripheral blood parameters and cellularity in celastrol treated-BALB/c mice. (A) Peripheral blood parameters in celastrol treated-mice. Mice received four consecutive daily IP injections of DMSO 5% or various concentrations of celastrol (0.01, 0.1, 1 or 5 mg/kg/day). Peripheral blood was harvested the day following the last injection. Values are percent of control. RBC: red blood cells; HGB: Hemoglobin; HCT: hematocrit; WBC: white blood cells. Mean ± SEM, n = 10. (B) Cellularity in control and celastrol treated-mice. Mice received four consecutive daily IP injections of DMSO 5% or celastrol (5 mg/kg). Cells from bone marrow (1 femur+1 tibia), spleen and peritoneal cavity were harvested the day following the last injection. Mean ± SEM, n = 10.

To investigate the effects of celastrol on the hematopoietic system, mice received daily IP injections (0.01, 0.1, 1 or 5 mg/kg/day) over the course of 4 days, and tissues were harvested the day following the last injection. Analysis of PB revealed similar counts of red blood cells and similar levels of hemoglobin and hematocrit in mice treated at all concentrations of celastrol, compared with control mice treated with 5% DMSO (Figure 1A). Mice treated with the highest concentration of celastrol showed signs of toxicity with hunched posture and ruffled fur. These mice appeared to have more total white blood cells than did control mice, although it was not statistically significant (n = 10; p>0.05) (Figure 1A). This data indicated that we should further analyze mice treated with celastrol at 5 mg/kg/day.

### 2- Celastrol treatment results in multiple defects in mature lineages

**Figure 2:**
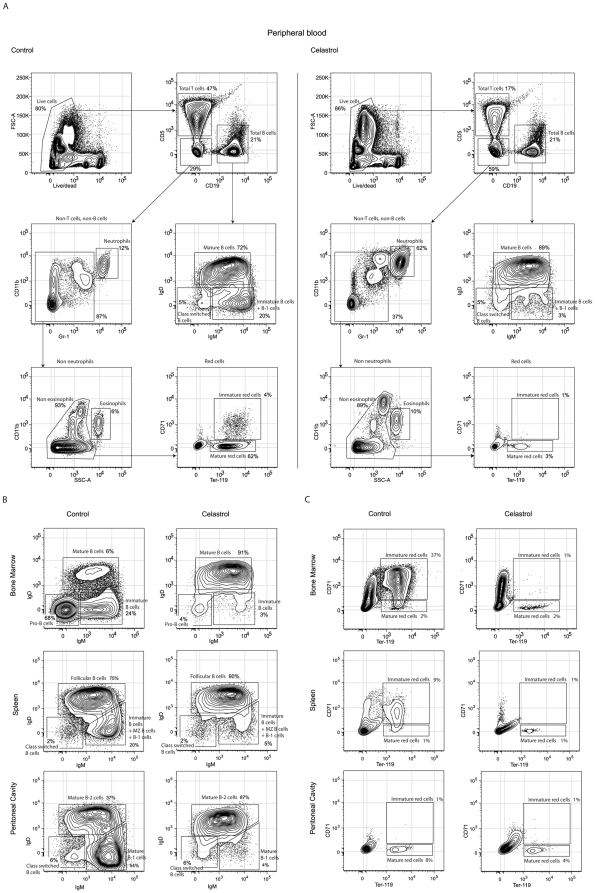
Celastrol treatment results in multiple defects in mature lineages. (A) Representative FACS analysis of mature lineages from peripheral blood of control (left) and celastrol treated-mice (right). Mice received four consecutive daily intraperitoneal injections and peripheral blood cells were harvested the day following the last injection, processed and analyzed as described in Materials and Methods. The percentages shown correspond to raw data numbers. Data shown are representative of 10 mice. (B) Representative FACS analysis of total B cells (gated as LIVE/DEAD^+^, CD5^−^, CD19^+^, and analyzed for IgD and IgM expression) from bone marrow, spleen and peritoneal cavity of control (left) and celastrol treated-mice (right) (n = 10). FACS-analysis of the different sub-populations of B lymphoid progenitors was performed as described [13], [20]. MZ B cells: Marginal Zone B cells. (C) Representative FACS analysis of total red cells (gated as LIVE/DEAD^+^, CD5^−^, CD19^−^, CD11b^−^, Gr-1^−^, SSC-A^low^ and analyzed for CD71 and Ter119 expressions) from bone marrow, spleen and peritoneal cavity of control (left) and celastrol treated-mice (right) (n = 10). FACS-analysis of the different sub-populations of red cell progenitors was performed as described [21], [22].

We then examined the distribution of the different PB hematopoietic lineages using flow cytometric analysis and specific cell surface markers. The percentages shown correspond to raw data numbers. FACS analysis revealed dramatic alterations in the lymphoid, myeloid and erythroid populations following celastrol treatment (Figure 2A), in agreement with previous studies describing aplastic anemia in patients treated with extracts of *T. wilfordii* [14], and blood defects in zebrafish embryos exposed to pure celastrol [15]. Lymphoid markers indicated that blood from celastrol treated-mice contained fewer total T lymphocytes (CD5^+^) (2.8-fold; *p<0.05) with no apparent change in the total B lymphocyte population (CD19^+^). Interestingly, celastrol induced a significant decrease in the number of immature B and B-1 cells (6.7-fold; ***p<0.001) with a modest increase in the mature B cell population (1.2-fold; **p<0.01) (Figure 2A). Among the remaining “non-T” and “non-B” PB cells, the cell size combined with staining for Gr-1 and CD11b myeloid markers indicated a significant increase of the neutrophils (5.2-fold; ***p<0.001) and a slight increase of the eosinophils (1.7-fold; **p<0.01) (Figure 2A). This may be a consequence of an inhibition of the infiltration of inflammatory cells into tissues since celastrol has been previously shown to reduce the total number of inflammatory cells in peribronchial areas in a mouse asthma model [2]. The immature red cells, identified by the staining intensities of anti-Ter119 and -CD71 surface markers, were present but at an extremely low level (Figure 2A). These effects were transient, as recovery was complete four weeks after removal of the drug (data not shown).

The alterations affecting the development of B cells and erythrocytes were also found in BM as well as in spleen and PerC (Figure 2B and 2C). In the PerC, the B-1 population [13] was highly affected (13.5-fold decrease; ***p<0.001) (Figure 2B).

Celastrol did not appear to be directly cytotoxic in our study, as we did not observe decreases in the cellularity in any of the tested organs (Figure 1B). This is in agreement with a study describing no decrease in cell viability and no evidence of increased apoptosis of CD34^+^ human BM cells treated with extract of *T. wilfordii* [16]. Moreover, using colony-forming cell assays, it was demonstrated that the extracted compound directly blocks the ability of very early human hematopoietic multilineage, as well as lineage-specific committed, human hematopoietic progenitor cell to respond to growth factors and form colonies [16]. Thus we anticipate that the erythrocytic and B-lymphoblastic suppression described in our present study may be due to a loss of B and red cell regenerative potential from pluripotent cells exposed to celastrol.

### 3- Celastrol treatment results in multiple defects in bone marrow progenitors

**Figure 3:**
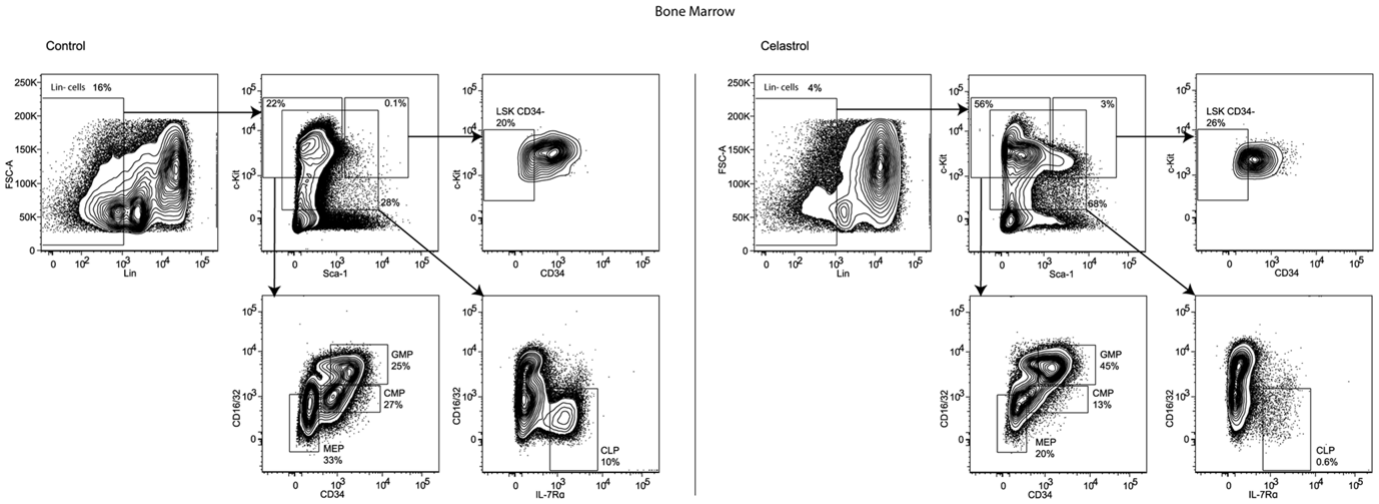
Celastrol treatment results in multiple defects in BM progenitors. Representative FACS analysis of Megakaryocyte-Erythrocyte Progenitors (MEP), Common Myeloid Progenitors (CMP), Granulocyte-Monocyte Progenitors (GMP), LSK CD34^−^ (Lin^−^ Sca-1^+^ c-Kit^+^ CD34^−^) cells and Common Lymphoid Progenitors (CLP) from bone marrow of control (left) and celastrol treated-mice (right) (n = 10). Cells were harvested from animals treated as described in Figure 2 and percentages shown correspond to raw data numbers. FACS-analysis of the different sub-populations of multipotent progenitors was performed as described [23].

Therefore, we examined the distribution of the different progenitors in the BM from celastrol treated-mice (Figure 3). We observed a 1.3-fold increase in the number of LSK CD34^−^ cells (Lin^−^ Sca1^+^ c-Kit^+^ CD34^−^), when compared to control mice (**p<0.01). We also showed decreases in the number of Common Myeloid Progenitors (CMPs) (2-fold; **p<0.01) and Megakaryocyte-Erythrocyte Progenitors (MEPs) (1.7-fold; **p<0.01), whereas the number of Granulocyte-Monocyte Progenitors (GMPs) increased (1.8-fold; **p<0.01). This suggests a potential priming of celastrol-treated CMPs towards GMPs. Finally, the number of Common Lymphoid Progenitors (CLPs) was significanly decreased (17-fold; ***p<0.001) after celastrol treatment.

These results show that celastrol specifically impairs the development of B cells and erythrocytes in PB, BM, spleen and PerC. Thus, a potential use of the drug could be to modulate the hematopoietic cell subsets, by treating cells ex vivo prior to adoptive transfer, or by treating donors prior to collection and purification of cells. Additional studies are needed to determine the kinetics of these effects, and to identify the proper dose of celastrol for each type of cell to be modulated.

The molecular mechanism underlying the effects of celastrol on the hematopoietic system is not well understood. One explanation may be the inhibition of Nuclear Factor-kappa B (NF-κB) pathways by celastrol [2]. A complete understanding of NF-κB signaling in erythropoiesis is not completely defined, but a role for NF-κB family members, including p105/p50, p100/p52 and p65, has been suggested [17], [18]. Moreover, genetic studies show that the different NF-κB proteins are important at different stages of B cell maturation [19]. Inhibition of NF-κB is a strategy that has great potential as a drug target in the treatment of various inflammatory diseases and cancer. Use of celastrol as a single agent or in combination with existing therapies for inhibition of NF-κB, may be an effective strategy. The multifaceted effects of celastrol on the hematopoietic system suggest that there may be several molecular targets, and this will need to be resolved for a more complete understanding of both the desired, and the adverse, effects of celastrol as a potential therapeutic agent. It is now apparent that the adverse effects of celastrol on the hematopoietic system need to be thoroughly evaluated prior to the initiation of further clinical studies.
